# Up-Beat UK: A programme of research into the relationship between coronary heart disease and depression in primary care patients

**DOI:** 10.1186/1471-2296-12-38

**Published:** 2011-05-23

**Authors:** André Tylee, Mark Ashworth, Elizabeth Barley, June Brown, John Chambers, Anne Farmer, Zoe Fortune, Mark Haddad, Rebecca Lawton, Anthony Mann, Anita Mehay, Paul McCrone, Joanna Murray, Morven Leese, Carmine M Pariante, Diana Rose, Gill Rowlands, Alison Smith, Paul Walters

**Affiliations:** 1 Health Services and Population Research Dept., Institute of Psychiatry, King's College London, De Crespigny Park, London, SE5 8AF, UK; 2 Department of Primary Care and Public Health Sciences, King's College London, 9th Floor, Capital House, 42 Weston Street, London, SE1 3QD, UK; 3 Department of Psychology, Institute of Psychiatry, King's College London, De Crespigny Park, London, SE5 8AF, UK; 4 Department of Cardiology, Guy's and St Thomas' Hospitals, Westminster Bridge Road, London, SE17EH, UK; 5 Department of Social Genetic and Developmental Psychiatry, Institute of Psychiatry, King's College London, De Crespigny Park, London, SE5 8AF, UK; 6 Department of Psychological Medicine, Institute of Psychiatry, King's College London, De Crespigny Park, London, SE5 8AF, UK; 7 Faculty of Health and Social Care, London South Bank University, 103 Borough Road, London, SE1 0AA, UK

## Abstract

**Background:**

Coronary heart disease and depression are both common health problems and by 2020 will be the two leading causes of disability worldwide. Depression has been found to be more common in patients with coronary heart disease but the nature of this relationship is uncertain. In the United Kingdom general practitioners are now being remunerated for case-finding for depression in patients with coronary heart disease, however it is unclear how general practitioners should manage these patients. We aim to explore the relationship between coronary heart disease and depression in a primary care population and to develop an intervention for patients with coronary heart disease and depression.

**Methods/design:**

This programme of research will consist of 4 inter-related studies. A 4 year prospective cohort study of primary care patients with coronary heart disease will be conducted to explore the relationship between coronary heart disease and depression. Within this, a nested case-control biological study will investigate genetic and blood-biomarkers as predictors of depression in this sample. Two qualitative studies, one of patients' perspectives of treatments for coronary heart disease and co-morbid depression and one of primary care professionals' views on the management of patients with coronary heart disease and depression will inform the development of an intervention for this patient group. A feasibility study for a randomised controlled trial will then be conducted.

**Discussion:**

This study will provide information on the relationship between coronary heart disease and depression that will allow health services to determine the efficiency of case-finding for depression in this patient group. The results of the cohort study will also provide information on risk factors for depression. The study will provide evidence on the efficacy and feasibility of a joint patient and professional led intervention and data necessary to plan a definitive randomised controlled trial of the intervention.

## Background

The World Bank and World Health Organisation have predicted that by 2020, coronary heart disease (CHD) and depression will be the two top causes of global health burden and disability[1]. As well as causing personal suffering, depression is a major public health problem responsible for 100 million lost working days in England and Wales at a cost of £9 billion per annum [2].

In the United Kingdom (UK), depression is one of the commonest reasons for consulting a general practitioner (GP). Up to 17% of consecutive GP attendees suffer from depression [3]. In the UK, 95% of people suffering from depression are treated solely in primary care [4].

Depression has been found to be more common in patients with coronary heart disease (CHD) with a prevalence of 20% [5]. Depression also increases the incidence and recurrence of acute coronary syndromes and death in patients with heart disease [5]. This increase in cardiac risk is independent of other risk factors for CHD [5]. The reason for the increased morbidity and mortality is unclear but may be due to increased activity of the immune/inflammatory pathways in depression, activating atherogenesis and plaque formation [6].

Depression is associated with a 50% increase in the costs of long term medical care after controlling for the severity of the physical illness[7]. Some of these costs are related to the association between depression and adverse health risk behaviours such as smoking, diet, lack of exercise and a lack of adherence to self-care regimes. Depression as cause or consequence of physical illnesses such as CHD may exacerbate the perceived severity of symptoms and with an increase in health service utilisation[7]. Treating depression and improving outcomes for depression has been shown to reduce health costs in people with physical illness[7,8]. It therefore seems important to address the care of patients with coronary heart disease and co-morbid depression in the primary care setting.

In the UK, GPs are now remunerated for depression case-finding in patients with CHD [9]. This is because evidence from two large trials in the United States has provided an indication in *post hoc *sub-group analyses that there may be cardiovascular benefits from treating depression using antidepressant medication[10,11]. Mortality seems to have been reduced in those whose depression improved and in those who took sertraline [12,13]. However, the natural history, morbidity and mortality of depression in the primary care population of patients with CHD are not known. It is also unclear how primary care professionals should manage additional cases of depression that may be identified in their CHD patients. Observational cohort studies are needed to help determine the effect of policy implementation on the patient's cardiac and depression outcomes as well as the effects of co-morbidity on morbidity and mortality. If the incidence of depression in patients with CHD is not solely a function of the current physical status, a cohort study will enable other factors associated with the incidence of depression e.g. factors relating to quality of life, illness attribution and social support, to be determined.

There are a number of treatment options for managing depression in primary care e.g. antidepressant medication, supervised exercise, guided self help, problem solving, computerised cognitive behavioural therapy (cCBT), group or individual CBT or interpersonal therapy (IPT) [4]. However the treatment preferences of patients with CHD are unknown. Primary care professional treatment preferences for managing depression in this population are also unknown. As with most physical illness in primary care settings, it is also unclear where the boundary exists between distress secondary to the physical condition and depression that would benefit from treatment. Qualitative studies with patients and their primary care professionals are needed to explore this further. A recent working party on the management of depression in CHD concluded that randomised controlled trials of stepped depression care versus treatment as usual for patients with CHD and depression are needed [5]. This programme of research will inform a randomised controlled trial of the effectiveness and cost-effectiveness of a case management approach to manage primary care patients with coronary heart disease and co-morbid depression. Case management has been shown to improve outcomes for depression in primary health-care settings [14], but there has been no research to determine whether it is effective in patients with symptomatic coronary heart disease. Case management has been defined as 'taking responsibility for following-up patients; determining whether patients were continuing the prescribed treatment as intended; assessing whether depressive symptoms were improving; taking action when patients were not adhering to guideline based treatment or were not showing expected improvement'[15]. It consists of five essential components [14]:

1. identification of patients in need of services

2. Assessing individual patient's needs

3. Developing a treatment plan

4. Coordination of care

5. Monitoring outcomes and altering care when favourable outcomes are not achieved

A randomised controlled trial is necessary to determine whether case management for this population is more effective than treatment as usual both in terms of depression and cardiac outcomes. A pilot randomised controlled trial is needed first to inform the design of the definitive randomised control trial.

This protocol describes a programme of research to develop and test a stepped care intervention for depression and coronary heart disease in primary care. It consists of 4 inter-related studies.

1. A 4 year cohort study of patients with coronary heart disease

2. A qualitative study of patients with coronary heart disease and co-morbid depression treatment preferences

3. A qualitative study of general practitioners (GPs) and practice nurses (PNs) treatment preferences for this patient group

4. A pilot randomised controlled trial of a case management programme for depressed primary care patients with CHD.

The first three studies will inform the development of the pilot randomised controlled trial (study 4). As the four studies are closely related and the randomised controlled trial depends on the conduct and findings of the other studies, the study protocols will be described in turn.

## Study 1: Cohort Study

### Objectives

The objectives of the cohort study are to:

• Determine prevalence, incidence rate and risk factors of depression in primary care patients with CHD

• Explore and describe the course, relationship, prognosis and current management of physical and depressive symptoms in primary care patients with CHD and co-morbid depression over a four-year period.

• To determine the effect of co-morbid depression on mortality, symptom severity, quality of life, disability, pain, service use (at all levels) and service costs, and lost employment costs in primary care patients with CHD.

•In a subsample, to investigate genetic variation and blood biomarkers that characterise depression in this cohort.

### Hypotheses

In addition to the estimation of prevalence and incidence of depression among patients with CHD, the main hypotheses to be tested in this study are:

• That symptoms and disability caused by CHD are associated with depression and that in patients with symptomatic CHD, the severity of symptoms of CHD, is a greater predictor of developing a depressive episode than a past history of depression.

• That in patients with symptomatic CHD, the severity of depressive symptoms as measured by the Hospital Anxiety and Depression Scale (HADS) [16] is related to the severity of CHD symptoms as measured by the Rose Angina Questionnaire [17] and SF-36[18].

• That patients with CHD and depression use more services for the management of their CHD, are more costly, and have higher lost employment costs than non-depressed primary care patients with CHD.

#### Methods/design

This is a naturalistic cohort study in primary care in South London, United Kingdom. Eligible consenting patients will be assessed at baseline and then at 6 monthly intervals for up to 4-years.

#### Study Population

Patients with CHD will be recruited from practice CHD registers. Two subgroups will be identified:

1. Patients with CHD and a current diagnosis of depression

2. Patients with CHD who are not currently depressed

#### Recruitment of study population

GPs are remunerated for keeping CHD registers under the Quality and Outcomes Framework. Practices participating in this Framework and based in South London will be recruited by the Greater London Research Network (GLRN). All patients on the CHD registers in participating general practices will be invited to participate in the study. Consenting patients will be assessed at baseline and then every 6 months over a 4 year period.

#### Measures

Information on sociodemographic status, past medical and psychiatric history, prescribed medication and medication compliance will be collected. Measures used at baseline and follow-ups [17-31] are shown in Table 1.

**Table 1:** Measures that will be used at baseline and follow-up assessments

Measure	Baseline	Follow-up
		

Modified Rose Angina Questionnaire [17]	x	x

Guy's Hospital Chest Pain Questionnaire [19]	x	x

Specific Activity Scale[20]	x	x

General Health Questionnaire-12 (GHQ-12_ [21]	x	

Hospital Anxiety and Depression Scale (HADS)[22]	x	x

Clinical Interview Schedule-Revised (CIS-R) [23]	x	

Patient Health Questionnaire-9 (PGQ-9) [24]	x	x

EuroQoL (EQ-5D) [25]	x	x

Medical Outcomes Survey Short Form-12 (SF-12) [18]	x	x

List of Threatening Experiences Questionnaire [26]	x	x

Social Problems Questionnaire (SPQ) [27]	x	x

Client Service Receipt Inventory (CSRI) [28]	x	x

Brief Illness Perceptions Questionnaire (B-IPQ) [29]	x	x

Psychological Outcome Profiles Questionnaire (PSYCHLOPS) [30]	x	x

Rapid Estimate of Adult Literacy in Medicine (REALM) [31]	x	

In addition, for the nested biological study we will investigate "at risk" genetic polymorphisms in neurotransmitters and immune genes (for example, serotonin transport or interleukin-6) and inflammatory biomarkers (for example, C-reactive protein and cortisol).

#### Inclusion criteria

• Currently registered with GP

• On the coronary heart disease register of participating general practitioners

• Over 18 years of age

#### Exclusion criterion

• Patients only temporarily registered with participating practices

#### Sample size calculation

The national prevalence of CHD in primary care as estimated by the Quality and Outcomes Framework (QOF) disease registers is 3.5%. We estimate that in patients with CHD, 20% will have a diagnosis of depression.

In a practice population of 10,000 people, we would therefore expect 350 patients with CHD of which 70 would be currently be depressed. Three practices with an average population of 10,000 patients will be recruited initially. Allowing for 80% participation, this will provide ~ 670 patients with CHD but no depression and ~168 patients with CHD and co-morbid depression. A cohort of this size would be large enough to estimate rates to acceptable limits on the basis of the above figures. The total sample size (~800) would produce up to 4800 data points over 4 years of follow-up at 6-monthly intervals (6 measures per person). This total sample size will be effectively somewhat smaller, firstly because of attrition and secondly because of the lack of independence of the repeated measures. Assuming an intra-person correlation of 0.2, the sample size would reduce by approximately 1/3 and if 20% dropout is also assumed, a total sample size of ~3130 data points would be collected, large enough to allow modelling of the associations between physical and mental illness as specified in the hypotheses.

#### Analysis

A descriptive analysis of prevalence and incidence, course, prognosis and management of physical and depressive symptoms will be undertaken. Risk and rate ratios will be estimated for the effect of depression on: symptom severity, quality of life, functional status, pain, and service use. An economic evaluation will be conducted to establish economic burden. Multiple regression using methods for repeated measures will be used to explore the relationship between depression, CHD and other possible confounding factors using the longitudinal data. The time course will be examined by including time as a main effect and as an interaction with the physical illness. The relationship of depression severity to outcome measures will also be explored using multiple regression analyses.

Service costs will be calculated by combining service use data from the Client Service Receipt Inventory with appropriate unit cost information. Lost employment costs will be estimated by multiplying lost workdays by daily wage rates. Regression models will be used to identify patient characteristics that explain variations in costs. Due to the expected skewed distribution of the cost data we will use bootstrapping and generalised linear models. It would be expected that service inputs over time (represented by service costs) would influence clinical measures and quality of life. To assess this we will also use regression models with symptoms and quality of life as dependent variables and the costs of specific services entered as independent variables, along with socio-demographic covariates.

#### Time Scale

The cohort study will run for up to 4 years. The Programme is a five-year study, the first 6 months will be spent obtaining the necessary ethics and research governance approvals and the final year will be spent analysing the data, writing up and dissemination. The cohort study will therefore, in effect, run for three and a half years allowing at least 6 follow-up data points after the initial baseline assessments supposing that patients are enrolled into the cohort by 12 months from the start of the study.

#### Ethical approval

This study was granted ethical approval by the Bexley and Greenwich Research Ethics Committee (REC Reference: 07/H0809/38)

## Study 2: A Qualitative Study of patients' perceptions of distress and depression in patients with CHD

### Aims

The second study in the Up-Beat Programme is a qualitative study of the perceptions of depression and distress in patients with CHD who have significant depressive symptoms. The aim of this study is to elicit patients' perceptions of their psychological state as linked to their CHD and explore their views on appropriate treatments for distress or depression in the context of their CHD. A second aim is to explore particular distinctions they may make between distress and depression.

### Sample

Up to fifty people will be purposively sampled from the cohort study based on age, gender, practice, CHD status, and depression severity.

### Method/design

Consenting participants will have a semi-structured interview partly informed by an initial focus group of patients with symptoms of depression and co-morbid CHD. The interview will also cover:

• A description of the participant's current psychological state

• What treatments they think are appropriate for depression/psychological distress?

• How participants may prevent or take protective measures in the context of both CHD and distress/depression

• The views of participants on medication for both their heart condition and depression

### Analysis

The interviews will be audio-taped and transcribed verbatim. The analysis will be assisted by computer software, namely, NVivo7 (QSR International, 2006). A thematic analysis of the data will be conducted. The themes will be guided by the data but there will be two *a priori *areas of investigation; firstly the links made by the participant between their physical condition and their psychological state; secondly which treatments, if any, they think are appropriate? Discourse Analysis will be used to explore how participants think about their psychological state and to examine the language they use. Their use of language will be used to help develop a scale to differentiate between distress and depression as below.

### Developing a scale to differentiate distress and depression

A pilot scale, based on the analysis above, will be constructed that can be used to differentiate between distress and depression in primary care clinical practice. Focus groups will be convened to assess the validity of the scale. The scale will be factor analysed to see if there is a two-factor solution - one for distress and one for depression. The correlation of the scale, and sub-scales with a quantitative measures of depression will be assessed. A merged factor analysis on both the quantitative and patient-generated scales together will then be undertaken.

#### Ethical approval

This study was granted ethical approval by the Bexley and Greenwich Research Ethics Committee (REC Reference: 07/H0809/38)

## Study 3: A Qualitative Study of health professionals' perceptions of distress and depression in patients with CHD

### Objectives

The objectives of the third study in the Up-Beat programme are:

1. To explore primary care professionals' views on distress and depression in patients with CHD

2. To explore their current management strategies and attitudes to a range of treatments in relation to this patient group

3. To provide guidance on the design and implementation of a practice-nurse led case-management depression intervention.

### Method/design

Individual in-depth interviews will be conducted with up to 20 general practitioners and practice nurses in primary care centres in South London. The sample will be selected purposively to include professionals working in different settings (single-handed and group practices) serving areas of contrasting socio-economic, gender and ethnic characteristics. Potential participants will be invited to take part in an interview with the aim of exploring primary care professionals' perceptions of distress and depression in patients with coronary heart disease. An initial interview guide will be developed from the research literature. Key topics to be explored in the interviews are likely to include:

• Observations on the impact of distress and depression on help-seeking in patients with CHD

• Perceptions of the presentation of distress/depression in people with CHD, including patients' beliefs and attributions

• Age, gender, ethnic and cultural differences in the above

• Vulnerability and risk factors for depression in patients with CHD, including social and economic factors

• Current management strategies, including primary and secondary care interventions, patient self-care and non-medical or "life-style" strategies

• Attitudes to various treatment options, including practice-nurse led case-management

Interviews will be recorded on audiotape and transcribed verbatim. The interviewing and analysis procedure will be based on grounded theory principles in which theories are generated from themes identified in the analysis of participant interviews [32]. At least two researchers will read the transcripts of early interviews several times to immerse themselves in the data and then independently identify and give a descriptive "code" to relevant sections of the text. They will then compare their coding and any discrepancies will be discussed and resolved. An iterative procedure is followed in which new themes emerging from interviews are added to the coding frame and earlier transcripts recoded. New themes will be added to the interview guide to be explored in later interviews. The computer programme NVivo 7 (QSR International, 2006) will be used to process the transcripts, code themes systematically and explore the associations between them.

### Using key findings to inform the development of an intervention

Key findings from the interview study will be used in informing the choice and content of the pilot intervention for the randomised controlled trial using the following approach:

Choice of intervention

• Findings and recommendations from qualitative studies 2 and 3 will be assessed by an intervention working group, consisting of members of the research team and steering group. These will then inform the content and delivery of the case management intervention.

#### Patient participation via two focus groups

• A purposive sample of 8-10 qualitative interview participants will be selected to take part in 2 consecutive, focus groups, using the same sample for each group.

• The aim of the first group is to discuss the proposed pilot intervention and elicit suggestions for additions and/or alterations to the proposed content.

• The aim of the second group is to re-present the amended pilot intervention content and to elicit final comments and minor alterations that may be needed.

### Ethical approval

This study was granted ethical approval by the Bexley and Greenwich Research Ethics Committee (REC Reference: 07/H0809/38)

## Study 4: Feasibility study for a randomised controlled trial of case management for depression in primary care patients with symptomatic coronary heart disease

### Objectives

The objectives of this pilot are:

1. *Clinical efficacy of case management*

To explore whether case management for primary care patients with symptomatic coronary heart disease and depression, when delivered by mental health professionals may be more effective than treatment as usual.

2. *Sample size calculation*

Estimates of the location of the mean and variability around the mean (standard deviation) for the primary outcome measure (see measures) will be calculated. A conservative estimate using the 95% upper confidence limit will be used to inform the sample size calculation of the definitive RCT.

3. *To enable selection of the most appropriate primary and secondary outcome measures*. The pilot will also allow potential secondary outcome measures to be assessed.

4. *Integrity of the study protocol*

The pilot will allow all procedures for a definitive effectiveness RCT to be piloted. This will include testing:

• inclusion/exclusion criteria

• training of staff in the administration and assessment of the intervention.

5. *Testing data collection forms and questionnaires*

This will ensure that the questionnaires are acceptable to the participants, are comprehensible, appropriate, clearly defined and presented in a consistent manner. Patient information documents and consent forms will also be tested. Inter-rater reliability between researchers will be tested.

6. *Randomization procedure*

The randomisation process and acceptability of randomisation to primary care professionals and participants will be tested.

7. *Recruitment and consent*

The recruitment method will be tested and the consent rate for participants into the study calculated. Barriers to recruitment of both practices and participants will be explored. Follow-up rates will be calculated.

8. *To determine the acceptability of the intervention and the trial to practices and participants*. To determine the possible sources of contamination, and to develop a standardised manual for case management for use in the definitive RCT. To make an informal assessment of the degree to which the intervention can be standardised and whether therapist effects are likely to be a major factor.

### Method/design

This will be a pilot randomised controlled trial conducted in primary care. A descriptive account of the process and participants will be recorded.

#### Setting and Practice recruitment

The study will be carried out in primary care. Practices in South London which are not participating in the cohort study will be recruited via the Greater London Primary Care Research Network (PCRN-GL) and invited to participate in the pilot study. We estimate from the results of the cohort study that we will need 10-15 practices each with around 10,000 patients in order to randomise between 30 and 50 participants per arm.

#### Participant recruitment

All patients on practice case registers for CHD will be asked by their GP for consent to contact from a researcher. Those consenting will be contacted by a researcher and assessed for depression using the Patient Health Questionnaire-2[33] and for symptoms relating to CHD using the Modified Rose Angina Questionnaire [34]. Patients scoring 3 or more on the PHQ-2, and with symptomatic CHD will then be assessed further using the Hospital Anxiety and Depression Scale (HADS)[22]. If they score >9 on the depression scale of HADS they will be eligible to participate in the study. Those consenting to participate will then be randomised to either to the intervention (case management) or the treatment as usual (TAU) arm of the study.

#### Sample size

If we want to show a mean difference between intervention and control of ≥ 3 on the HADS Depression subscale, assuming a standard deviation around mean scores of 4, we will need 30 participants per group for 90% power at the 95% significance level. Therefore 60 participants (30 per arm) will be recruited into the RCT.

#### Inclusion Criteria (Practices)

• Practice keeps a register of patients with CHD for the Quality and Outcomes Framework (QOF) and is willing to liaise over patients in the case management arm when necessary

#### Inclusion Criteria (Participants)

• Symptomatic CHD as scored on the modified Rose Angina Questionnaire

• A score ≥8 on the depression part of the Hospital Anxiety and Depression Scale

• Aged 16 years or over

#### Exclusion Criteria (Participants)

• Temporary registrations

• Actively suicidal patients

• Psychotic depression as evidenced by delusions and/or hallucinations

• Non-English speaking

• Participants currently in hospital for treatment of their CHD

Up to 20 group practices of around 10,000 patients each from South London will be recruited by the Greater London Local Research Network (GLLRN) to participate in the study.

#### Intervention

The nature of the intervention will be determined by the results of the two qualitative studies.

#### Control

Participants randomised to the TAU control group will receive treatment as usual by their GP and any other relevant professionals.

#### Measures

Participants will be followed up at 1-, 6- and 12-months post randomisation. The same measures will be applied at each follow-up. The measures to be used are shown in Table 2.

**Table 2:** Measures to be used at each time point in the pilot randomised controlled trial

Outcome Parameter	Instruments
	

*Primary Outcome*	

Depression	Hospital Anxiety and Depression Scale[22]

*Secondary Outcome*	

Depression	Patient Health Questionnaire-9 [24]

Coronary Heart Disease	Rose Angina Questionnaire[17], Guy's Hospital Chest pain Questionnaire[19], Specific Activity Schedule [20]

Quality of Life	Euroqol-5D [25], Medical Outcomes Survey Short Form-12 (SF-12) [18]

Adherence to medication	Adapted version of Morisky adherence questionnaire[35]

Life events	List of Threatening Events Questionnaire [26]

Social problems	Social Problems Questionnaire [27]

Health Service Utilisation	Client Service Receipt Inventory (CSRI) [28]

Illness Perceptions	Brief Illness Perceptions Questionnaire [29]

Wellbeing	Warwick-Edinburgh Mental Well-being Scale [36]

Participants problem priorities	PSYCHLOPS [37]

In order to evaluate the process of delivering the intervention, focus groups of participants in the intervention arm and primary care professionals will be conducted to explore their experiences, views on the intervention, and their experience of participating in this study.

#### Randomisation

The unit of randomisation will be the participant. Randomisation will be conducted independently by the Clinical Trials Unit at the Institute of Psychiatry, King's College London. Randomisation will be by random permuted blocks.

#### Blinding

Researchers will be blind to randomisation status. Researchers will be asked to give their opinion on randomisation status at the end of the study to determine whether blinding was adequate.

#### Statistical analysis

Descriptive analyses will be used to provide summary estimates of outcome measures, focussing on the dropout rate at each time point, the order of magnitude of the effect of the intervention and the variability of the outcome measure at baseline. T-tests and regression modelling will be used to test whether there is a statistically significant difference between intervention and control groups. All analyses will be by intention to treat. While the sample size will not be sufficient to test clustering effects formally, sources of clustering will be identified so that they can be taken into account in a definitive RCT.

### Ethical considerations

Ethical approval has been granted by the South East London Research Ethics Committee (REC Ref No. 10/H0808/51).

Participants may withdraw from the study at any time and without giving any reason without their care being affected. Any participant thought to be suicidal, either by the researchers (as assessed on depression questionnaires), or by the case managers will have their GP informed immediately. Case managers will have regular supervision by clinicians experienced in general practice and psychiatry employed on the research team. The team also has an experienced consultant cardiologist to provide advice where appropriate.

## Discussion

We have described the rationale and protocol for a programme of research into coronary heart disease and depression in primary care. This programme has been funded and is currently underway. This programme of research should provide important data to help improve the management of primary care patients with co-morbid CHD and depression. As far as we are aware this will be the first research into coronary heart disease and depression in the primary care population. This is important as in the United Kingdom the majority of patients with depression are treated entirely in primary care. A strong point in the design of this research is that the cohort study will be large enough and conducted over a long enough period of time to be able to explore potential associations between depression and coronary heart disease and to determine morbidity and mortality in this patient group. The nature of the intervention will take into account patient perspectives as well as health professional perspectives. This should allow the development of an intervention that also addresses the concerns of patients. A potential limitation of the research is that it is difficult to estimate recruitment rates into the cohort study as these vary widely in the literature. A low recruitment rate could mean that our cohort is not representative of the general practice population with coronary heart disease and depression. We will however test how representative our cohort is by comparing it with routinely collected general practice data from within the same catchment area as the study cohort.

## Competing interests

The authors declare that they have no competing interests.

## Authors' contributions

All authors participated in the conception and design of this research programme including the randomized controlled trial. AT, PW, ML and PM drafted the protocol for the cohort study. PW, AT and ML drafted the protocol for the trial. DR, JM, EB, AT and PW drafted the protocols for the 2 qualitative studies. MA, JB, JC, MH helped with the development of the protocols and provided content specific expertise for the study protocols and development of the study materials. All critically revised the manuscript. All authors read and approved the final manuscript.

## Pre-publication history

The pre-publication history for this paper can be accessed here:

http://www.biomedcentral.com/1471-2296/12/38/prepub
